# Surgical Ritual

**Published:** 1896-11-07

**Authors:** 


### Surgical Ritual.

It is no doubt a sign of much superiority to speak lightly of the ritual of one's faith. Nevertheless, it is apt to have a bad influence on weak-kneed believers, and although, as a mere subject for banter and for more or less humorous description, the "uncomfortable extravagances" of some Continental operating theatres readily lend themselves to the sarcasms of the practical man, we may well hesitate to endorse the ridicule recently thrown by a well-known London surgeon upon various antiseptic methods in common use abroad—methods which, after all, are but the carrying to a logical conclusion of principles now generally accepted by scientific surgeons all the world over, principles, in fact, which underlie all modern practice, even the practice of the surgeon we refer to.

Mr. Treves, speaking at Reading the other day, made great fun of "the glass table for the patient, of the exquisite ceremonial of washing on the part of the operator, of the rites attending the ostentatious cleansing of the patient, of the surgeon in his robes of white mackintosh and his indiarubber fishing boots, and of the onlookers beyond the pale who are excluded, with infinite solicitude, from the sacred circle as septic outlaws," and no doubt the practical surgeon, operating in all sorts of houses, and still meeting with success, may be strongly led to look upon such refined aids to surgical cleanliness as more allied to "a fervent idolatrous ritual" than as having any place in surgery. It must, however, be admitted that such aids can do no harm unless, as Mr. Treves suggests, the attention of the surgeon is so concentrated on the "ritual" that the surgery itself is lost sight of. He says, "At present the actual handling of knives and forceps is overwhelmed by the dexterous disposal of the wash basin and the soap dish." But is this so? Is it a fact that Continental surgeons, and, we might add, some English surgeons also, are so snatching at the shadow of antiseptic purity as to be losing sight of the reality of true surgical principles? This may well be doubted. Both on the Continent and in America a splendid record of good work is being built up by those who, while endeavouring to carry the war against septic infection to its extreme logical conclusion, are at the same time behind none of their colleagues in boldness, dexterity, and surgical acumen.

It will be admitted at once that the elaborate precautions which are taken in some operating theatres are not essential, and that by the use of strong chemical disinfectants a good average of success may be obtained. It may even be admitted that for outside work in private practice aseptic surgery in the modern sense may prove to be impossible, and chemical antiseptics may be the only road to such success as is attainable.

If, however, we are to accept aseptic surgery as our ideal it is difficult to deny that in institutions where work of all kinds is being done by all kinds of operators, the attainment and maintenance of asepsis in an operating theatre must be infinitely facilitated by the appliances being so constructed as to be capable of being readily cleansed; that in a hospital which cannot pick and choose its cases, a careful, even though apparently elaborate, method of cleansing the patient before the operation is desirable; and that in regard to the cleansing of the surgeon himself a little "ritual" is no bad thing, even if it do nothing more than demonstrate to the surgeon which of his assistants he may safely trust.

But, in fact, the "ritual" complained of is but the routine which has been found by a considerable experience to be efficient for bringing the average man into a state of surgical cleanliness. Some men require much less of this surgical "grooming" than is necessary for others, but when it is a question of teaching, as it is in our large hospitals, the average man only should be considered. In describing the antiseptic method he adopts, Mr. Treves says, "The surgeon is clean, but he does not parade his cleanness." This is where we venture to differ from him. It is admitted that the cleanliness of the surgeon is the key to his success, and the surgeon who steps into the theatre spick and span, and, although clean, makes no parade of his cleanness, just fails to teach the students the very trick on which the life of the patient hinges. This "parade" may be laughed at and spoken of as ritual; but in surgery, as in religion, ritual is not all parade, being symbolical of many things which it is well to have constantly recalled to mind.

